# Prediction of Postoperative Pathologic Risk Factors in Cervical Cancer Patients Treated with Radical Hysterectomy by Machine Learning

**DOI:** 10.3390/curroncol29120755

**Published:** 2022-12-06

**Authors:** Zhengjie Ou, Wei Mao, Lihua Tan, Yanli Yang, Shuanghuan Liu, Yanan Zhang, Bin Li, Dan Zhao

**Affiliations:** 1 Department of Gynecology Oncology, National Cancer Center, National Clinical Research Center for Cancer, Cancer Hospital, Chinese Academy of Medical Sciences, Peking Union Medical College, Beijing 100021, China; 2 Department of Gynecology Oncology, The Fifth People’s Hospital of Qinghai Province, Xining 810007, China

**Keywords:** blood biomarker, cervical cancer, deep stromal infiltration, lymph node metastasis, lymph-vascular space invasion, machine learning methods

## Abstract

Pretherapeutic serological parameters play a predictive role in pathologic risk factors (PRF), which correlate with treatment and prognosis in cervical cancer (CC). However, the method of pre-operative prediction to PRF is limited and the clinical availability of machine learning methods remains unknown in CC. Overall, 1260 early-stage CC patients treated with radical hysterectomy (RH) were randomly split into training and test cohorts. Six machine learning classifiers, including Gradient Boosting Machine, Support Vector Machine with Gaussian kernel, Random Forest, Conditional Random Forest, Naive Bayes, and Elastic Net, were used to derive diagnostic information from nine clinical factors and 75 parameters readily available from pretreatment peripheral blood tests. The best results were obtained by RF in deep stromal infiltration prediction with an accuracy of 70.8% and AUC of 0.767. The highest accuracy and AUC for predicting lymphatic metastasis with Cforest were 64.3% and 0.620, respectively. The highest accuracy of prediction for lymphavascular space invasion with EN was 59.7% and the AUC was 0.628. Blood markers, including D-dimer and uric acid, were associated with PRF. Machine learning methods can provide critical diagnostic prediction on PRF in CC before surgical intervention. The use of predictive algorithms may facilitate individualized treatment options through diagnostic stratification.

## 1. Introduction

Cervical cancer remains one of the most frequent malignant tumors in women [1]. With the widespread application of human papillomavirus (HPV) vaccination and the popularity of screening, patients diagnosed at early stages have accounted for the majority. Radical hysterectomy (RH) is the standard-of-care treatment for these patients [2]. The unavoidable problem after surgery is whether adjuvant treatment is required, which is judged in accordance with postoperative pathological risk factors. The likelihood of risk factors that increase the risk of recurrence is high, especially in stage IB3-IIA2 (the 2018 International Federation of Gynecology and Obstetrics, FIGO) due to large tumor bulk [2]. Previous studies have illustrated that neoadjuvant chemotherapy (NACT) plus surgery inhibited micro-metastasis and distant metastasis of tumors, and was associated with a declined incidence of pathologic risk factors [3]. However, despite the fact that NACT reduces the rate of adjuvant therapy after surgery, patients treated with NACT cannot be thoroughly free from radiotherapy and the adverse effects that radiotherapy brings. 

In addition, concurrent chemoradiotherapy (CCRT) is also an alternative initial treatment for early-stage cervical cancer, particularly for locally advanced cervical cancer. As for a patient with several pathologic risk factors, conformed to the adjuvant therapy standard, CCRT should be considered as the initial therapy but not RH, which shortens the treatment process for the same effect and reduces treatment costs [4]. With regard to patients staged ⅠB-ⅡA, according to the National Comprehensive Cancer Network (NCCN) guidelines, concurrent chemoradiation and RH both serve as alternative primary treatment options, sharing nearly therapeutic equivalence. However, increased morbidity and complications have been specifically illustrated when surgery and radiotherapy are combined [5,6]. This multimodal treatment modality has caused them to bear a double treatment burden and increased medical cost. In addition, the successive therapeutic process also prolongs the treatment period, aggregates their side effects and affects quality of life in the long run. Accordingly, it is necessary to construct a model to predict pathologic risk factors before primary treatment, which will help select those for whom it is more appropriate to receive direct chemoradiation therapy rather than RH. Additionally, the development of model to predict postoperative pathologic risk factors is an important element for individual prognosis stratification and personalized medicine. 

Pathologic risk factors in cervical cancer include lymph node metastasis (LNM), parametria infiltration, positive surgical margins, lymph-vascular space invasion (LVSI), tumor size >4 cm and deep stromal infiltration (DSI) [2]. Previous studies illustrated that many clinicopathologic factors were related to pathologic risk factors by common statistical methods, but these methods were not suited to handle more complex data [7,8,9]. Machine learning is a branch of artificial intelligence (AI) technology that allows the computer to conclude potential rules from complicated data of retrospective examples. AI technology has been widely used to analyze clinical material to construct a model to predict clinicopathological factors and treatment outcome, acquiring a properly higher accuracy compared with traditional statistical methods [10,11,12]. Therefore, it is feasible and reasonable to apply machine learning to the prediction of postoperative pathologic risk factors. 

Based on the successful application of AI technology and the discovery of related factors with pathologic risk factors, we hypothesized that pretreatment of clinicopathological factors would be effective in the prediction of postoperative pathologic risk factors by machine learning analysis in FIGO stage IB-IIA cervical cancer. In addition, because of the low incidence rate of positive margins and parametria infiltration in primary cohorts and preoperative confirmation of tumor size via clinical palpation, this study’s outcome contained a prediction of other pathologic risk factors. Therefore, in the present study, we aimed to explore the construction of a model for predicting LNM, LVSI and DSI through machine learning combing of clinicopathological biomarkers and explore unreported significant parameters associated with these factors.

## 2. Materials and Methods

### 2.1. Patients and Considered Features

This was a retrospective cohort study of 1260 patients with FIGO stage (2003) IB and IIA cervical cancer who were treated with RH with retroperitoneal lymphadenectomy between 2003 and 2017 in our institution (National Cancer Center/Cancer Hospital, Chinese Academy of Medical Sciences; CICAMS). We retrospectively collected clinicopathological parameters, including age at diagnosis, body mass index (BMI), menopausal status, clinical FIGO stage, gross type, histologic grade, clinical tumor diameter, 75 preoperative peripheral blood biomarkers, etc. (Table 1 and Table S1). Tumor diameter was obtained via clinical palpation before surgical intervention. 

### 2.2. Data Splitting

We obtained 1260 samples after preliminary preprocessing: removing medically impossible data (containing obvious record error), removing the features with 10% missing values and the samples with missing values. Variables of age, BMI, menopausal status, clinical tumor diameter, histology, FIGO stage, gross type, previous abdominal surgery, histologic grade (obtained via cervical biopsy preoperatively) and 75 pretreatment peripheral blood markers were all incorporated into the model construction. We started to handle the features: the continuous features were normalized and categorical features were one-hot coded, and LinearSVC method with L1 penalty was used to choose features.

The dataset was split into training and test cohorts according to a ratio of 1:1 by repeated random sampling until there was no significant difference (*p* value > 0.05) between the two cohorts with respect to the three tasks (Table 1). The *p* values were calculated using Chi-square or Fisher exact test for categorical variables, and the student’s *t*-test or the Mann–Whitney U test were conducted for analyzing normally distributed or non-normally distributed continuous variables. This resulted in the training cohort and the test cohort both having 630 patients. 

### 2.3. Supervised Machine Learning Classifiers

In this study, we evaluated six types of supervised machine learning classifiers, including GBM (Gradient Boosting Machine) [13,14], SVMRadial (Support Vector Machine with Gaussian kernel) [15], RF (Random Forest) [16], Cforest (Conditional Random Forest) [17], NB (Naive Bayes) [18] and EN (Elastic Net) [19]. In addition, a logistic regression classifier was used as a baseline. R software version 4.2.1 with R package caret was used to implement all classifiers. One hundred independent training sets were conducted using different random seeds in order to calculate variable importance for prediction. We used the median of variable importance acquired from each training as a representative value. The importance of each variable was calculated using the varImp function of the caret package. A RF classifier combines two machine learning techniques: bagging and random feature selection consisting of a group of decision trees. Cforest is an algorithm using conditional inference trees as base learners, implementing both the random forest and the bagging ensemble algorithm. EN is a logistic regression classifier trained by using a regularized method that linearly combines the L1 and L2 penalties of the lasso and ridge methods. 

### 2.4. Model Assessment

To assess the performance of different models, we computed the accuracy (ACC) and the area under the ROC curve (AUC) on the test cohort as our evaluation metrics. Here, ACC was obtained by setting the threshold corresponding to the top left point of the ROC curve. As the AUC is independent of the chosen threshold, we used it as the main evaluation metric.

### 2.5. Confidence of Prediction and Shannon’s Information Gain

Shannon’s information gain was used to assess the prediction confidence [20]. If a patient, *i*, is lacking the information concerning the class that the patient is included in (k-class), the Shannon’s information entropy representing uncertainty is expressed with:

$$H(i)=\log_{2}k$$

If a classifier provides prediction probabilities for each class, the entropy will be:

$$H_{c}(i)=\sum_{j=1}^{k}p_{j}(i)\log_{2}(p_{j}(i))$$

Here, $p_{j}(i)$ is the predicted probability that the patient *i* is included in class *j*. Thus, we obtain the information gain, i.e., information gained by the prediction:

$$IG(i)=H(i)-H_{c}(i)$$

The individual information gain for each class is given by:

$$IG_{j}(i)=p_{j}(i)\times IG(i)$$

## 3. Results

### 3.1. Prediction of Deep Stromal Infiltration of Cervical Cancer Based on Multiple Preoperative Blood Markers Using Machine Learning Methods

**Figure 1:**
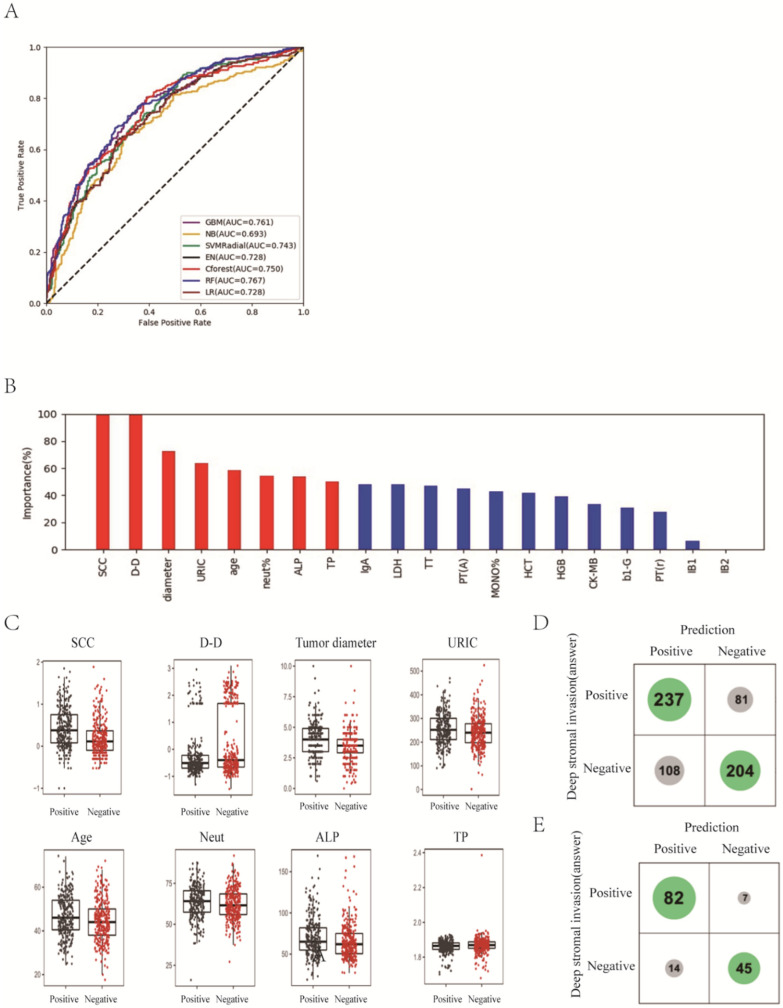
Prediction of deep stromal infiltration of cervical cancer based on multiple preoperative blood markers using machine learning methods. (**A**) ROC curves derived from logistic regression for predicting deep stromal infiltration of cervical cancer based on all 75 peripheral blood markers using machine learning methods compared with logistic regression. (**B**) Relative importance of variables for prediction of deep stromal infiltration calculated in the RF. Variable importance is represented as a percentage of the highest value. (**C**) Box and jitter plots representing the distribution of top eight important parameters for distinguishing infiltration from non-infiltration. (**D**,**E**), Confusion matrix indicating the prediction quality of the RF classification for all predictions (**D**) and for those predictions with high (>0.2 bits) confidence (**E**). Notes: SCC, squamous cell carcinoma antigen; D-D, D-dimer; URIC, uric acid; ALP, alkaline phosphatase; TP, total protein; IgA, immunoglobulin A; LDH, lactate dehydrogenase; TT, thrombin time; PT(A), plasma prothrombin time ratio (A); MONO%, percentage of monocytes; HCT, hematocrit; HGB, hemoglobin; CK-MB, creatine kinase-MB isoenzyme; b1-G, beta 1 globulin; PT(r), plasma prothrombin time ratio (r).

Depth of stromal invasion was evaluated by an experienced pathologist and was recognized as significant, with more than one millimeter of invasion in the depth of the stroma in a microscopic examination. The status of the depth of stromal infiltration was classified into two groups: “non-deep” and “deep”. The “deep” group referred to patients who had an invasive carcinoma with greater than one-third stromal invasion according to the pathologic findings. “Non-deep” indicated a carcinoma infiltrating no more than one third of the cervical stroma. The values for the highest ACC of the prediction and the AUC were 70.8% and 0.767 with RF classifier, which achieved a 5.4% higher score than the traditional method of multiple logistic regression analysis in AUC (Figure 1A; Supplemental Table S2). It is notable that the best two classifiers, RF and GBM, both used ensemble methods that combine weak decision trees.

Next, we focused on the best model, RF, and understood the variables. The relative importance of each variable for segregating deep stromal infiltration patients from non-deep infiltration ones was calculated for RF (Figure 1B). We identified the top eight factors, including SCC, D-D, tumor diameter, URIC, age, neut%, ALP and TP, as important RF predictors for distinguishing deep infiltration from non-deep infiltration. Standard box plots that presented the distribution of each variable between deep and non-deep samples are shown in Figure 1C. 

Interestingly, we found that D-D was a critical variable, in addition to SCC. From the confusion matrix (Figure 1D), RF predicted 81 patients with deep infiltration as ones with non-deep infiltration and predicted 108 patients with non-deep infiltration as ones with deep infiltration. When we considered the Shannon gain to represent the confidence of predictions and chose those patients with certain higher confidence of predictions, the predictions designated as higher confidence (>0.2 bits from Shannon information gain computation) contained only 21 mispredictions out of 148 instances (Figure 1E). In particular, for the predictions with higher confidence, if a patient was predicted as non-deep, this was right at a rate of 1 − 7/52 = 86.5%.

### 3.2. Differentiation of Lymph Node Metastasis of Cervical Cancer with Machine Learning Methods

**Figure 2:**
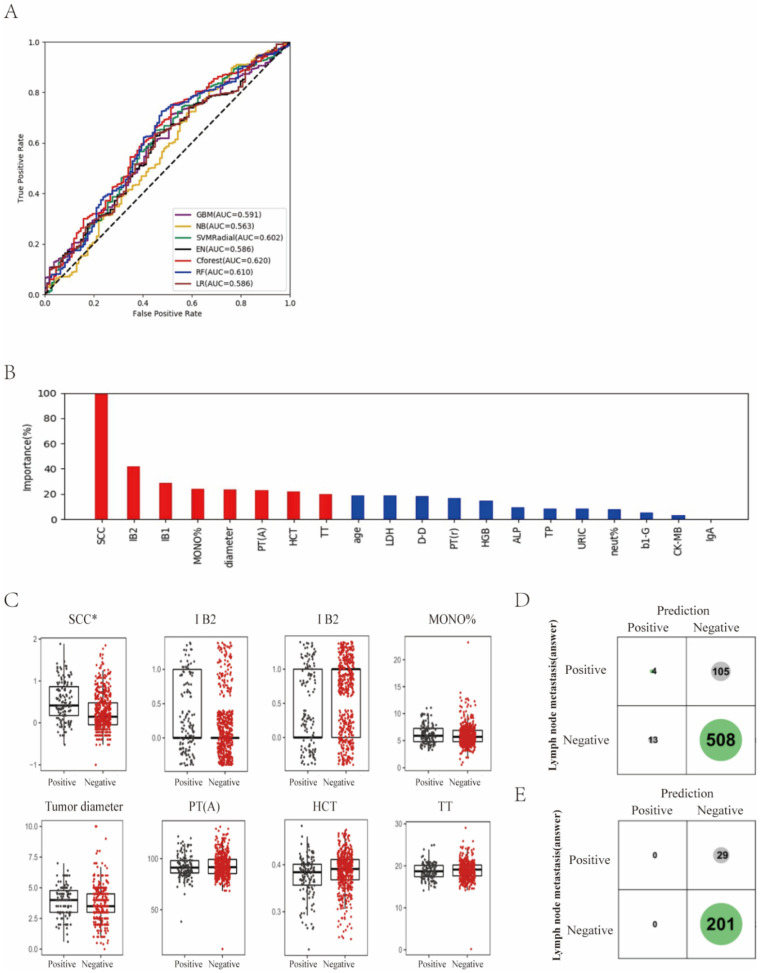
Differentiation of lymph node metastasis of cervical cancer with machine learning methods. (**A**) ROC curves derived from logistic regression for predicting lymph node metastasis of cervical cancer based on all 75 peripheral blood markers using machine learning methods compared with logistic regression. (**B**) Relative importance of variables for prediction of lymph node metastasis calculated in the Cforest. Variable importance is represented as a percentage of the highest value. (**C**) Box and jitter plots representing the distribution of top eight important parameters for distinguishing metastasis from non-metastasis. (**D**,**E**), Confusion matrix indicating the prediction quality of the Cforest classification for all predictions (**D**) and for those predictions with high (>0.2 bits) confidence (**E**). Notes: SCC, squamous cell carcinoma antigen; MONO%, percentage of monocytes; PT(A), plasma prothrombin time ratio (A); HCT, hematocrit; TT, thrombin time; LDH, lactate dehydrogenase; D-D, D-dimer; PT(r), plasma prothrombin time ratio (r); HGB, hemoglobin; ALP, alkaline phosphatase; TP, total protein; URIC, uric acid; neut%, percentage of neutrophils; b1-G, beta 1 globulin; CK-MB, creatine kinase-MB isoenzyme; IgA, immunoglobulin A.

The status of lymph node metastasis was classified into two groups: “metastasis” and “non-metastasis”. We found that Cforest showed the best prediction performance with an ACC of 64.3% and an AUC of 0.620 (Figure 2A; Supplemental Table S2), which achieved a 5.8% higher score than LR in AUC.

Next, the relative importance of a variable for segregating metastatic patients from non-metastatic ones was calculated for Cforest (Figure 2B). We identified the top eight factors, including SCC, IB2, IB1, MONO%, diameter, PT(A), HCT and TT, as important Cforest predictors for distinguishing metastatic patients from non-metastatic ones. It should be noted that as the clinical stage progresses, SCC and tumor diameter can increase. Standard box plots that presented the distribution of each variable between metastatic and non-metastatic samples are shown in Figure 2C.

**Figure 3:**
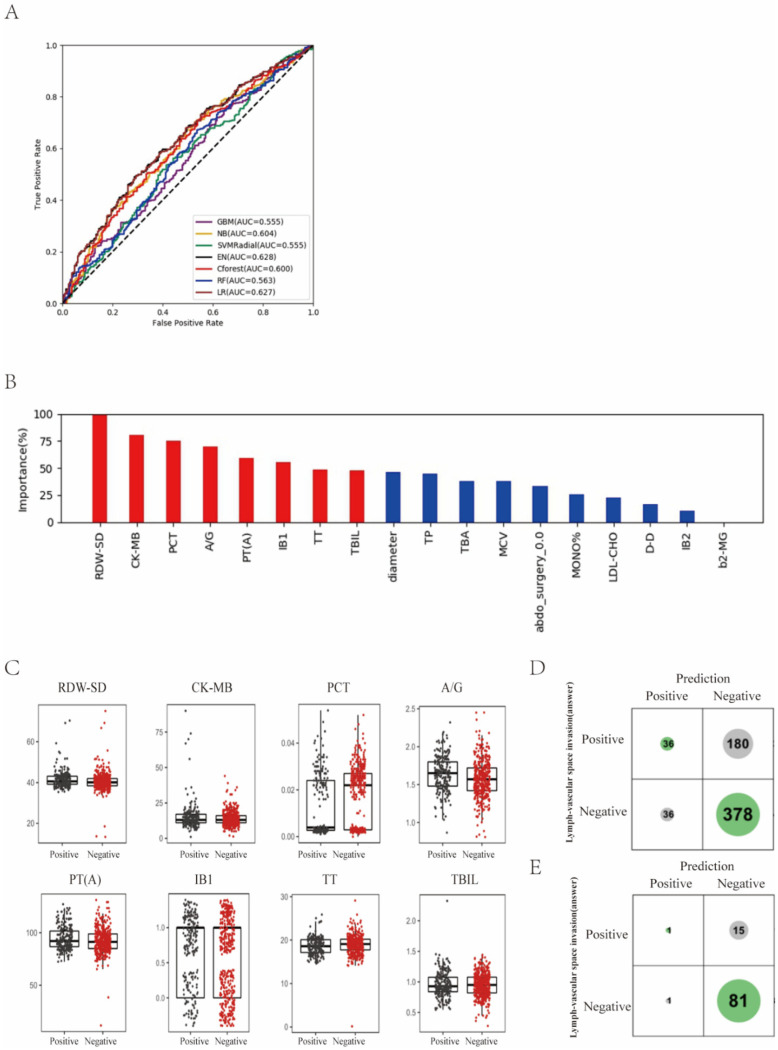
Prediction of lymph-vascular space invasion of cervical cancer based on preoperative blood markers using machine learning methods. (**A**) ROC curves derived from logistic regression for predicting lymph-vascular space invasion of cervical cancer based on all 75 peripheral blood markers using machine learning methods compared with logistic regression. (**B**) Relative importance of variables for prediction of lymph-vascular space invasion calculated in the EN. Variable importance is represented as a percentage of the highest value. (**C**) Box and jitter plots representing the distribution of top eight important blood markers for distinguishing invasion from non-invasion. (**D**,**E**) Confusion matrix indicating the prediction quality of the EN classification for all predictions (**D**) and for those predictions with high (>0.2 bits) confidence (**E**). Notes: RDW-SD, standard deviation of red blood cell distribution width; CK-MB, creatine kinase-MB isoenzyme; PCT, plateletcrit; A/G, albumin to globulin ratio; PT(A), plasma prothrombin time ratio (A); TT, thrombin time; TBIL, total bilirubin; TP, total protein; TBA, total bile acid; MCV, mean corpuscular volume; abdo_surgery_0.0, previous abdominal surgery; MONO%, percentage of monocytes; LDL-CHO, low density lipoprotein cholesterol; D-D, D-dimer; b2-MG, beta 2 microglobulin.

Interestingly, we found that SCC was a critical variable. From the confusion matrix (Figure 2D), RF predictions had 105 false negative samples and 13 false positive samples. However, predictions designated as higher confidence (>0.2 bits from Shannon information gain computation) contained only 29 misprediction out of 230 instances (Figure 3E). In particular, for the predictions with higher confidence, if a patient was predicted as non-metastasis, this was right at a rate of 1 − 29/230 = 87.4%.

### 3.3. Prediction of Lymph-Vascular Space Invasion of Cervical Cancer Based on Preoperative Blood Markers Using Machine Learning Methods

In the task of lymph-vascular space invasion, patients were labeled as “invasion” or “non-invasion”. LVSI refers to the presence of epithelial tumor cells in the lumen of vessels. “Invasion” indicated positive pathologic findings of LVSI and “non-invasion” indicated no pathologic proof of LVSI. We found that EN showed the best prediction performance, with ACC of 59.7% and AUC of 0.628, and the traditional method of multiple logistic regression analysis was comparative with ACC of 59.5% and AUC of 0.627 (Figure 3A; Supplemental Table S2). 

Next, the relative importance of each variable for segregating invasion from non-invasion was calculated for EN (Figure 3B). We identified the top eight factors, including RDW-SD, CK-MB, PCT, A/G, PT(A), IB1, TT and TBIL, as important EN predictors for distinguishing invasion patients from non-invasion ones. Standard box plots that present the distribution of each variable between invasion and non-invasion are shown in Figure 3C.

Interestingly, we found that RDW-SD was a critical variable. From the confusion matrix (Figure 3D), EN predictions had 180 false negative samples and 36 false positive samples. However, predictions designated as higher confidence (>0.2 bits from Shannon information gain computation) contained only 15 misprediction out of 98 instances (Figure 3D,E). In particular, for the predictions with higher confidence, if a patient was predicted as non-invasion, it was right at a rate of 1 − 15/98 = 84.7%.

## 4. Discussion

In recent years, machine learning algorithms based on AI technology have been widely accepted and extensively utilized for diagnostic and prognostic assessment of various types of cancers in the context of precision medicine [11,21,22]. This innovative approach, serving as an important tool with high accuracy and efficient ability to process complex data, can explore the key related factors to effectively assist in the clinical decision making of cervical cancer treatment. More importantly, hidden and embedded patterns within familiar clinical data can be revealed with the aid of AI models. However, so far, no studies have been conducted on integrating readily accessible clinical blood markers into the model construction of predicting pathologic risk factors in cervical cancer based on AI technology. Our study allowed for the comparison of various machine learning algorithms with the traditional logistic regression analysis to identify the approach with the most favorable performance and explore the serologic biomarkers with potential diagnostic potency. In cervical cancer with FIGO stage IB-IIA, radical hysterectomy followed by tailored adjuvant radiotherapy and concurrent chemoradiotherapy are both recommended for suitable treatment modalities [21]. Postoperative adjuvant radiotherapy is warranted for women with histopathologically verified risk factors, such as LVSI, LNM, DSI, etc., to improve prognosis [22,23,24], which led to an increase in the risk of higher morbidity [25,26,27]. It is beneficial and meaningful to predict pathologic risk factors so as to identify those more likely to receive postoperative adjuvant radiotherapy to avoid compounding treatment-related morbidity. Currently, the lack of ability to accurately identify those with a higher chance to receive postoperative radiotherapy and achieve individualized medical management instead of a “one-size fits all” approach has been a primary clinical limitation. Therefore, predicting pathologic risk factors by comprehensive utility of laboratory blood tests and other pretreatment information is a fundamental way toward individualized optimal medical care. In this study, we explored the ability of multiple machine learning methods to predict pathologic risk factors of patients with cervical cancer by incorporating readily available blood biomarkers. We found that three ensemble classifiers, RF, Cforest and EN, were able to predict pathologic risk factors of early-stage cervical cancer, in which RF showed the best predictive performance with an appreciable accuracy of 70.8% and AUC of 0.767 for DSI. Cforest showed the most accurate predictive value for LNM (64.3% accuracy and 0.620 AUC), and EN for LVSI (59.7% accuracy and 0.628 AUC). Compared to the traditional approach of logistic regression analysis, the RF classifier achieved a 5.4% higher score of AUC in DSI prediction, Cforest achieved a 3.4% higher score of AUC in LNM prediction and EN showed almost the same performance in LVSI prediction. The underperformance of these classifiers with regard to LNM and LVSI may be attributable to the lack of particularly strong distinctions of cervical cancer at the level of an early stage based on serum biomarkers. Nevertheless, the results indicate that AI technology can provide valuable predictive information before primary treatment to facilitate individualized medical strategy. In addition, based on the optimal results of machine learning algorithms, this study may offer useful clinical information concerning variables that are of most importance for identification of pathologic risk factors, like DSI, in early-stage patients. 

Previous evidence has suggested that cancer is a metabolic disease associated with inflammation [28]. Cervical cancer harbors a unique collection of inflammatory and metabolic molecules in the serum [29]. In early-stage cervical cancer, local inflammatory processes may be at an initial state in which the peritumoral microenvironment perhaps alters the most, while distant and systemic metabolic features and cancer-target responses are immunosuppressed [30], leading to the slight distinction of cancer invasiveness, which was obscured in serum markers. Understandably, as tumor debulk progresses, tumor burden aggravates, leading to cancer invasiveness. In this study, we found that squamous cell carcinoma antigen (SCC), D-dimer and uric acid (UA) levels were the top five significant plasma biomarkers for predicting DSI. SCC has been considered as the most important diagnostic and prognostic tumor marker in cervical cancer. Many studies demonstrated that an elevated level of pretreatment serum SCC was closely associated with disease progression and recurrence [31,32]. UA is a powerful antioxidant and considered as a protective factor against cancer [33]. It has been reported that an elevated level of UA was associated with cancer risk, aggressiveness and poor oncologic outcomes in various cancer types [34,35,36], but few studies have focused on gynecologic cancer. Interestingly, previous studies have also shown a prooxidant role of UA [37] and lower levels of UA were associated with elevated risk of cancer-related mortality compared with high levels [38]. The precise relation of UA with cancer, especially cervical cancer, needs further study. D-dimer serves as a valuable marker of activation of coagulation and fibrinolysis, and is also known as a biomarker of cancer prognosis, especially in metastasized patients [39,40,41]. The pretreatment prediction model of DSI in cervical cancer performed well and revealed potential meaningful serum biomarkers that were readily available in clinical settings, which is also consistent with previous studies. This study’s findings suggest that the supervised machine learning analysis serves as a feasible and effective approach that can aid in discovering more meaningful biomarkers that are correlated with PRF in cervical cancer and are not identified by conventional multiple regression analysis. 

Identification of reliable pretreatment blood markers associated with pathologic risk factors helps clinicians in clinical decision making [42]. In this study, we found some serologic indicators, such as RDW-SD and other indicators, that had scarcely been found to be related to the diagnosis and prognosis of cervical cancer in previous studies. We found that RDW was the top predictive indicator for LVSI. RDW is a routinely measured hematological index, primarily reflecting the degree of anisocytosis. It has been reported that this simple and inexpensive parameter is a strong and independent risk factor for death in the general population [43]. Research has demonstrated that an aberrant elevation level of RDW leads to poor survival outcomes in most tumor types and stages, independent of age, gender or region [44]. However, little is known about RDW in cervical cancer. One recent study indicated that RDW was associated with worse prognosis in cervical cancer [45]. Excessive oxidative stress, inflammation, and cell senescence were proposed as the conditions that RDW associates closely with mortality [46,47]. More dataset analysis is still needed to confirm the predictive ability of these factors. Based on the high efficiency of pretreatment blood markers, the dynamic detection of serological indicators in multiple time periods may be more powerful in prediction. As the dynamic analysis of serological indicators is more complex, future studies should develop the use of artificial intelligence-based machine learning algorithms to identify the predictive features of preoperative blood variable time series, which might significantly facilitate the accuracy of clinical characteristics prediction and deserve further study. 

As tumors progress over time, the signal transduction and correlation between the tumor and its microenvironment, including fibroblasts, tumor-related immune cells and endothelial cells, will become increasingly closer [48]. The changes of peripheral blood parameters before surgery were inherently a combination of tumor-specific and microenvironment-specific factors and the result of the interaction between tumor and microenvironment. Given the importance of tumor microenvironment in the process of tumor development, clinicians should make full use of preoperative peripheral blood indicators for treatment decision making, cancer progression evaluation and prognosis assessment. In previous studies, clinicians often ignored the reflection of regular blood biomarkers on the biological characteristics of tumors and relied almost exclusively on tumor-specific factors as included indicators for assessment, which was also a common problem in previous retrospective analysis of tumors. In this study, we identified a series of blood indicators that were readily available and necessary for preoperative evaluation related to pathologic risk factors by machine learning methods, such as UA, D-dimer, thrombin time, AST, MONO%, RDW-SD, etc. These parameters have the potential to be related to the microenvironment in cancer progression or metastasis, and their changes will also influence treatment timing and selection.

There have been a few previous studies exploring the use of serologic biomarkers to predict PRF. One study [49] in 2016 incorporated clinical factors and three blood markers derived from pretreatment blood routine examination to predict LNM, patients’ overall survival and recurrence-free survival. They found platelet/lymphocyte ratio were significantly associated with LNM. Another study [50] in 2020 found that pretreatment albumin to fibrinogen ratio was significantly related to lymph node metastasis, depth of stromal infiltration, etc. Many studies focused on prediction for survival outcomes or a single PRF of cervical cancer based on clinical factors [51,52,53] and/or radiomic parameters [54,55]. However, no studies have made an attempt to predict three PRFs based on a series of clinically readily available blood markers. In addition to critical data analysis methods based on clinical factors, there are still many studies exploring new approaches of postoperative pathologic risk factors prediction. It is clear that the diagnosis of pathologic risk factors could only be accurately judged from the postoperative report of cervical cancer. Identification of reliable approaches that are able to predict pathologic risk factors in advance would facilitate the identification of more accurate diagnostic stratification and a more appropriate treatment strategy. A previous study indicated that DSI can be determined by combining the 2D or 3D ultrasound with clinical variables before treatment, with over 70% accuracy and AUC [56]. However, this diagnostic approach depended more on subjective judgment rather than objective parameters based on relatively few cases. It was reported that the assessment of cervical cancer with full-thickness stromal invasion by MRI examination was limited [57]. In Bidus’s study, the conical method combined with clinical factors to determine DSI and LVSI before treatment also achieved good accuracy but this method is a destructive examination and may easily interfere with the complete resection of radical surgery [58]. In the study of LNM diagnosis, sentinel node staining is currently the most commonly developed method, but it is only used to determine whether complete lymph node resection is performed before surgery [59,60]. In this study, LNM was associated closely with primary tumor size as staging and tumor diameter were among the top five predictors for LNM. Results indicated that imaging materials, such as MRI, reflecting the visual size of the tumor itself and enlarged lymph nodes would potentially provide more accurate predictive information preoperatively. However, previous studies also used magnetic resonance imaging (MRI) and ultrasound to determine lymph node metastasis, but imaging data could only determine lymphadenectasis rather than tumor cell metastases in most cases, which leads to the unsatisfactory accuracy of the prediction model [56,61]. This is a reminder that traditional data analysis on simple integration of imaging information is not adequate enough to achieve LNM prediction. It is promising to achieve more comprehensive and precise prediction by virtue of effective integration of high-throughput extraction of a large amount of information from images based on AI technology, which will be the focus of our subsequent research. As the approach used in this study did not consider any information from pretreatment biopsies or imaging studies, there may be a limitation of the ability to predict pathologic risk factors before initial treatment; indeed, more independent datasets from other institutions are required to investigate how pretreatment blood signatures can be utilized for more accurate assessment of pathologic risk factors. Manipulation of high-throughput sequencing analysis, such as RNA sequencing, of pretreatment peripheral blood may improve predictive performance, however, from another perspective, it may become more complicated and expensive to incorporate RNA analysis information into the process of preoperative assessment in the current context of clinical settings. Further comprehensive investigation is needed in the hope of achieving the best clinical and socioeconomic benefits.

Our study has some limitations. Firstly, this study was a single-center retrospective study. The retrospective nature may result in inherent bias. Secondly, results from our database should be supplemented with external and prospective validation for prevention of overfitting as well as further spread of application in clinical practice. Thirdly, other machine learning approaches should be undertaken to manage the missing data in future work. Fourthly, our assessment of diagnostic ability to predict pathological risk factors was preliminary, and further study is warranted to better validate the accuracy of blood biomarkers. At present, our model is not sufficiently powerful and accurate to predict LVSI and LNM, but some blood biomarkers have been revealed for the first time that may be potentially useful predictors from a large number of variables. However, a positive prediction is not trivial; compared with traditional methods, the machine learning algorithms could serve as a feasible tool for clinicians to predict oncologic outcomes based solely on pretherapeutic information. 

## 5. Conclusions

This study indicates that AI-based algorithms are useful tools that may aid in providing critical information for diagnostic evaluation of pathologic risk factors in patients with cervical cancer before initial treatment. The use of predictive algorithms may facilitate personalized treatment selection through pretherapeutic assessment.

## Figures and Tables

**Table 1:** Clinical and pathologic characteristics of 1260 patients with cervical cancer.

Variables	All Patients (*n* = 1260)	Training Cohort (*n* = 630)	Test Cohort (*n* = 630)	*p* Value
Age (years)	45 (18–74)	45 (18–74)	45 (21–73)	0.777
BMI (kg/m^2^)	23.6 (16.0–42.7)	23.6 (16.0–47.5)	23.7 (16.5–42.7)	0.453
Menopausal status				
Yes	353 (28.0%)	446 (70.8%)	461 (73.2%)	0.347
No	907 (72.0%)	184 (29.2%)	169 (26.8%)	
Clinical tumor diameter (cm)	3.5 (0.5–8.0)	3.5 (0.5–10.0)	3.5 (0.5–8.0)	0.211
Histology				
Squamous carcinoma	1053 (83.6%)	525 (83.3%)	528 (83.8%)	0.82
Adenocarcinoma	133 (10.6%)	69 (11.0%)	64 (10.2%)	0.647
Others	74 (5.8%)	36 (5.7%)	38 (6.0%)	0.811
FIGO stage (2003)				
IB1	707 (56.1%)	361 (57.3%)	346 (54.9%)	0.394
IB2	289 (22.9%)	142 (22.5%)	147 (23.3%)	0.738
IIA1	135 (10.7%)	60 (9.5%)	75 (11.9%)	0.172
IIA2	129 (10.3%)	67 (10.6%)	62 (9.8%)	0.642
Gross type				
Exophytic	1163 (92.3%)	587 (93.2%)	576 (91.4%)	0.245
Endophytic	97 (7.7%)	43 (6.8%)	54 (8.6%)	
Previous abdominal surgery				
Yes	255 (20.2%)	133 (21.1%)	122 (19.4%)	0.441
No	1005 (79.8%)	497 (78.9%)	508 (80.6%)	
Histologic grade				
Good	87 (6.9%)	43 (6.8%)	44 (7.0%)	0.912
Moderate	506 (40.2%)	256 (40.6%)	250 (39.7%)	0.73
Poor	667 (52.9%)	331 (52.5%)	336 (53.3%)	0.778
Deep stromal infiltration				
Negative	653 (51.8%)	335 (53.2%)	318 (50.5%)	0.338
Positive	607 (48.2%)	295 (46.8%)	312 (49.5%)	
Lymph-vascular space invasion				
Negative	829 (65.8%)	415 (65.9%)	414 (65.7%)	0.953
Positive	431 (34.2%)	215 (34.1%)	216 (34.3%)	
Lymph node metastasis				
Negative	1017 (80.7%)	496 (78.7%)	521 (82.7%)	0.074
Positive	243 (19.3%)	134 (21.3%)	109 (17.3%)	

## Data Availability

The datasets used and/or analyzed during the current study are available from the corresponding author on reasonable request.

## Supplementary Materials

The following supporting information can be downloaded at: https://www.mdpi.com/article/10.3390/curroncol29120755/s1, Table S1: Pretreatment peripheral blood tests of 1260 cervical cancer patients included in the primary cohort; Table S2: Diagnostic accuracy of clinicopathological factors using machine learning algorithms.
