# Supplementary material for: Prediction of Postoperative Pathologic Risk Factors in Cervical Cancer Patients Treated with Radical Hysterectomy by Machine Learning
Source: Curr Oncol. 2022 Dec 6;29(12):9613–29. doi: 10.3390/curroncol29120755 (PMC9776916; doi:10.3390/curroncol29120755)
Supplement: Supplementary file 1 [file curroncol-29-00755-s001.zip › curroncol-1985223-supplementary.pdf]

Supplementary Material

Table S1. Pretreatment peripheral blood tests of 1260 cervical cancer patients included in primary cohort.

Factors		Median	IQR		
WBC	G/L	6.33	5.32	-	7.72
neut%	%	63.10	56.80	-	69.18
NEUT	G/L	4.01	3.09	-	5.13
lymph%	%	28.40	23.03	-	34.40
lymph#()	G/L	1.77	1.45	-	2.15
MONO%	%	5.80	4.80	-	7.00
MONO#	G/L	0.37	0.30	-	0.46
EO%	%	1.30	0.70	-	2.30
EO#	G/L	0.08	0.04	-	0.15
baso%	%	0.30	0.20	-	0.50
baso#	G/L	0.02	0.01	-	0.03
RBC	T/L	4.39	4.12	-	4.65
HGB	g/L	132.00	123.00	-	139.00
HCT	L/L	0.39	0.37	-	0.41
MCV	fl	89.15	86.30	-	92.00
MCH	pg	30.10	28.90	-	31.10
MCHC	g/L	336.00	328.00	-	343.00
RDW-SD	fl	40.20	38.60	-	42.50
RDW-CV	%	12.50	12.00	-	13.10
PLT	G/L	250.00	212.00	-	296.75
MPV	fl	10.40	9.80	-	11.17
P-LCR	%	27.90	23.10	-	34.10
PDW	fl	11.90	10.70	-	13.48
PCT	L/L	0.02	0.00	-	0.03
K	mmol/L	4.24	4.03	-	4.47
Na	mmol/L	140.10	138.70	-	141.40
Cl	mmol/L	103.90	102.10	-	105.50
TCO2	mmol/L	24.50	23.00	-	26.00
ALT	U/L	14.00	11.00	-	20.00
AST	U/L	17.00	15.00	-	21.00
LDH	U/L	161.00	145.00	-	182.00
GGT	U/L	16.00	11.00	-	22.00
ALP	U/L	64.00	52.00	-	79.00
CK	U/L	61.00	48.00	-	78.00
CK-MB	U/L	13.00	11.00	-	17.00
GLU	mmol/L	5.23	4.88	-	5.60
TBA	umol/L	1.70	0.90	-	2.70
TBIL	umol/L	8.90	6.70	-	11.80
DBIL	umol/L	2.90	2.20	-	3.90
IBIL	umol/L	5.90	4.40	-	8.20
BUN	mmol/L	4.40	3.60	-	5.20
CRE	umol/L	57.00	51.00	-	63.00
URIC	umol/L	241.00	203.00	-	285.00
Ca	mmol/L	2.29	2.22	-	2.36
PHOS	mmol/L	1.17	1.07	-	1.27
Mg	mmol/L	0.88	0.83	-	0.92

CHOL	mmol/L	4.50	3.94	-	5.15
TG	mmol/L	1.02	0.75	-	1.45
HDL-CHO	mmol/L	1.38	1.17	-	1.62
LDL-CHO	mmol/L	2.83	2.31	-	3.39
β2-MG	ma/L	1.50	1.30	-	1.80
IgA	a/L	2.39	1.81	-	3.06
IgG	a/L	12.22	10.74	-	13.79
IgM	a/L	1.29	0.93	-	1.76
Fe	umol/L	13.50	9.10	-	18.38
TransFE	ma/dL	267.15	239.93	-	300.15
TP	a/L	73.70	70.50	-	76.80
ALB	a/L	45.40	43.40	-	47.10
G	a/L	28.10	25.80	-	30.78
A/G	/	1.61	1.45	-	1.75
PALB	ma/dL	24.00	21.00	-	28.00
LPa	ma/dL	3.70	3.40	-	4.20
α1-G	%	8.90	8.10	-	9.80
α2-G	%	6.00	5.60	-	6.50
β1-G	%	4.60	4.10	-	5.30
β2-G	%	17.70	16.00	-	19.50
γ-G	%	10.90	10.40	-	11.40
PT(A)	%	91.40	85.90	-	98.90
PT(r)	%	0.93	0.89	-	0.97
APTT	s	25.15	22.80	-	27.80
FIB	g/L	2.88	2.50	-	3.38
D-D	mg/L	0.34	0.21	-	1.28
FDP	ug/uL	0.80	0.30	-	1.40
TT	s	19.00	17.50	-	20.20
SCC	ng/uL	1.60	0.10	-	153.90

Notes: IQR, Interquartile range; WBC, White blood cell; neut%, Percentage of neutrophils; NEUT, Neutrophil count; lymph%, Percentage of lymphocyte; lymph#(), Lymphocyte count; MONO%, Percentage of monocytes; MONO#, Monocytes count; EO%, Percentage of eosinophils; EO#, Eosinophils count; baso%, Percentage of basophils; baso#, Basophils count; RBC, Red blood cell count; HGB, Hemoglobin; HCT, Hematocrit; MCV, Mean corpuscular volume; MCH, Mean corpuscular hemoglubin; MCHC, Mean cell hemoglobin concentration; RDW-SD, Standard deviation of red cell distribution width; RDW-CV, Variation coefficient of red cell distribution width; PLT, Platelet count; MPV, Mean platelet volume; P-LCR, Large platelet ratio; PDW, Platelet distribution width; PCT, Plateletcrit; K, Potassium; Na, Sodium; Cl, Chlorine; TCO2, Total carbon dioxide combining power; ALT, Alanine aminotransferase; AST, Aspartate aminotransferase; LDH, Lactate dehydrogenase; GGT, Gamma-glutamyl transpeptidase; ALP, Alkaline phosphatase; CK, Creatine kinase; CK-MB, Creatine kinase-MB isoenzyme; GLU, Glucose; TBA, Total bile acid; TBIL, Total bilirubin; DBIL, Direct bilirubin; IBIL, Indirect bilirubin; BUN, Blood urea nitrogen; CRE, Creatinine; URIC, Uric acid; Ca, Calcium; PHOS, Inorganic phosphorus; Mg, Magnesium; CHOL, Cholesterol; TG, Triglyceride; HDL-CHO, High density lipoprotein cholesterol; LDL-CHO, Low density lipoprotein cholesterol; β2-MG, Beta 2 microglobulin; IgA, Immunoglobulin A; IgG, Immunoglobulin G; IgM, Immunoglobulin M; Fe, Ferrum; TransFE, Transferrin; TP, Total protein; ALB, Albumin; G, Globulin; A/G, Globulin ratio; PALB, Prealbumin; LPa, Lipoprotein a; α1-G, Alpha 1 globulin; α2-G, Alpha 2 globulin; β1-G, Beta 1 globulin; B2-G, beta 2 globulin; γ-G, Gamma globulin; PT(A), plasma prothrombin time ratio(A); PT(r), Plasma prothrombin time ratio(r); APTT, Activated partial thromboplastin time; FIB, Fibrinogen; D-D, D-dimer; FDP, Fibrinogen degradation product; TT, Thrombin time; SCC, Squamous cell carcinoma antigen.

Table S2. Diagnostic accuracy of clinicopathological factors using Machine Learning Algorithms.

Methods	DSI		LNM		LVSI	
	**Accuracy (%)**	**AUC (95% CI)**	**Accuracy (%)**	**AUC (95% CI)**	**Accuracy (%)**	**AUC (95% CI)**
LR	0.683	0.728 (0.687-0.768)	0.619	0.586 (0.529-0.642)	0.595	0.627 (0.581-0.672)
SVMRadial	0.679	0.743 (0.703-0.782)	0.635	0.602 (0.544-0.659)	0.562	0.555 (0.507-0.602)
NB	0.671	0.693 (0.649-0.736)	0.640	0.563 (0.503-0.622)	0.605	0.604 (0.557-0.650)
RF	0.708	0.767 (0.729-0.804)	0.690	0.610 (0.552-0.667)	0.563	0.563 (0.515-0.610)
Cforest	0.700	0.750 (0.710-0.789)	0.643	0.620 (0.562-0.677)	0.600	0.600 (0.553-0.646)
GBM	0.703	0.761 (0.722-0.799)	0.594	0.591 (0.533-0.648)	0.565	0.555 (0.507-0.602)
EN	0.681	0.728 (0.688-0.767)	0.614	0.586 (0.539-0.632)	0.597	0.628 (0.582-0.673)

Notes: AUC, Area under the receiver operating characteristic curve; CI, confidence interval; LR, Logistic Regression; SVMRadial, Support Vector Machine with Gaussian kernel; NB, Naïve Bayes; RF, Random Forest; Cforest, Conditional Random Forest; GBM, Gradient Boosting Machine; EN, Elastic Net.
